# Arabidopsis MSI1 functions in photoperiodic flowering time control

**DOI:** 10.3389/fpls.2014.00077

**Published:** 2014-03-07

**Authors:** Yvonne Steinbach, Lars Hennig

**Affiliations:** ^1^Department of Biology, Institute of Agricultural Sciences, ETH ZürichZürich, Switzerland; ^2^Department of Plant Biology, Uppsala BioCenter, Swedish University of Agricultural Sciences and Linnean Center for Plant BiologyUppsala, Sweden

**Keywords:** Arabidopsis, flowering time, chromatin, MSI1, photoperiod, *FLOWERING LOCUS T* (*FT*), *CONSTANS* (*CO*)

## Abstract

Appropriate timing of flowering is crucial for crop yield and the reproductive success of plants. Flowering can be induced by a number of molecular pathways that respond to internal and external signals such as photoperiod, vernalization or light quality, ambient temperature and biotic as well as abiotic stresses. The key florigenic signal FLOWERING LOCUS T (FT) is regulated by several flowering activators, such as CONSTANS (CO), and repressors, such as FLOWERING LOCUS C (FLC). Chromatin modifications are essential for regulated gene expression, which often involves the well conserved MULTICOPY SUPRESSOR OF IRA 1 (MSI1)-like protein family. MSI1-like proteins are ubiquitous partners of various complexes, such as POLYCOMB REPRESSIVE COMPLEX2 or CHROMATIN ASSEMBLY FACTOR 1. In Arabidopsis, one of the functions of MSI1 is to control the switch to flowering. Arabidopsis MSI1 is needed for the correct expression of the floral integrator gene *SUPPRESSOR OF CO 1* (*SOC1)*. Here, we show that the histone-binding protein MSI1 acts in the photoperiod pathway to regulate normal expression of *CO* in long day (LD) photoperiods. Reduced expression of *CO* in *msi1*-mutants leads to failure of *FT* and *SOC1* activation and to delayed flowering. MSI1 is needed for normal sensitivity of Arabidopsis to photoperiod, because *msi1*-mutants responded less than wild type to an intermittent LD treatment of plants grown in short days. Finally, genetic analysis demonstrated that MSI1 acts upstream of the *CO-FT* pathway to enable an efficient photoperiodic response and to induce flowering.

## Introduction

The reproductive success of plants depends on the appropriate time to flower, which is of great agronomic relevance in crops. Flowering can be induced by a number of molecular pathways that respond to internal and external signals. Major genetic pathways controlling flowering time have been characterized based on the phenotype of *Arabidopsis thaliana* flowering time mutants in different growth conditions. These pathways include the photoperiod pathway, which responds to seasonal changes in day length, and the vernalization pathway, which responds to prolonged exposure to cold. The autonomous and gibberellin-pathways mediate the response to endogenous signals. Additionally, light quality, ambient temperature, and biotic as well as abiotic stresses can contribute to floral induction in plants (for review see: Jarillo and Piñeiro, 2011; Srikanth and Schmid, 2011).

The different pathways converge on pathway integrators, a set of genes that strongly promote flowering such as *FLOWERING LOCUS T* (*FT*), *SUPPRESSOR OF CONSTANS 1* (*SOC1/AGL20*) or the *FT* homolog *TWIN SISTER OF FT* (*TSF*). Mutants in these genes have late flowering phenotypes (Kardailsky et al., 1999; Kobayashi et al., 1999; Borner et al., 2000; Lee et al., 2000; Samach et al., 2000; Yamaguchi et al., 2005). The pathway integrators *FT* and *TSF* are antagonistically regulated by the floral repressor *FLOWERING LOCUS C* (*FLC*) (Michaels and Amasino, 1999) and the floral activator *CONSTANS* (*CO*) (Yanovsky and Kay, 2002).

The nuclear zinc finger transcription factor CO is the key activator in the photoperiod pathway to promote expression of *FT* and *TSF* (Suarez-Lopez et al., 2001; Valverde et al., 2004). CO protein is stable in the light and rapidly degraded in the dark. *CO* is regulated by the circadian clock and accumulates diurnally late in the day in long day (LD) conditions. In contrast, *CO* peaks during the night in SD where protein degradation prevents CO accumulation. *FT* is directly regulated by CO and follows the clock-regulated expression pattern of *CO* in LD (Suarez-Lopez et al., 2001; Valverde et al., 2004). Regulation of *FT* expression can occur also independently of *CO* and the photoperiodic pathway such as due to decreased red to far red light ratios in the shade avoidance response (SAR). SAR is mediated through the key regulator of the light-quality pathway phytochrom B by post-transcriptional repression of the *FT*-activator PHYTOCHROME AND FLOWERING TIME 1 (PFT1) (Cerdan and Chory, 2003; Halliday and Whitelam, 2003; Bäckström et al., 2007). In several species, such as Arabidopsis, tomato, tobacco and rice, the FT protein or its homologs are known to move from leaves into the shoot apical meristem (SAM) where it induces the switch to flowering (Corbesier et al., 2007) by inducing the expression of the downstream targets *SOC1* and *APETALA 1* (*AP1*) (reviewed in Zeevaart, 2008).

The floral integrators *FT*, *SOC1*, and *TSF* are commonly repressed by the potent flowering repressor *FLC*. Vernalization or the autonomous pathway of floral promotion establish low FLC levels and thus favor flowering. In contrast to other flowering time pathways, the autonomous pathway does not represent a linear genetic pathway and involves RNA-binding proteins (FCA, FPA, FLK), RNA processing proteins (FY) and chromatin regulators (FVE/MSI4, FLD) (for review see Simpson, 2004).

The autonomous pathway gene *FVE* is needed to establish repressive chromatin at *FLC* and encodes a MULTICOPY SUPRESSOR OF IRA 1 (MSI1)-like chromatin-adaptor protein (Ausin et al., 2004; Kim et al., 2004; Jeon and Kim, 2011). MSI1-like proteins belong to a subfamily of WD-40 repeat proteins that are subunits in several chromatin remodeling complexes (for review see Hennig et al., 2005). The single Drosophila MSI1-like protein p55 is a core subunit of Polycomb Group Repressive Complex 2 (PRC2), of Chromatin Assembly Factor 1, of histone deacetylase complexes and of other chromatin-associated protein complexes (for review see: Hennig et al., 2005). In contrast to flies, Arabidopsis has five MSI1-like proteins (MSI1-5). While Arabidopsis MSI4 was suggested to act in histone deacetylation (Ausin et al., 2004; Kim et al., 2004; Jeon and Kim, 2011), Arabidopsis MSI1 was shown to be part of Chromatin Assembly Factor 1 and PRC2-like complexes (Kaya et al., 2001; Köhler et al., 2003; Exner et al., 2006; Schönrock et al., 2006a; De Lucia et al., 2008; Derkacheva et al., 2013). MSI1 functions in the FERTILISATION INDEPENDENT SEED (FIS)-PRC2 complex, which silences target genes during gametophyte and early seed development (Köhler et al., 2003; Guitton et al., 2004; Guitton and Berger, 2005); the VERNALIZATION (VRN)-PRC2 complex, which is required for epigenetic repression of *FLC* and acceleration of flowering by extended cold (De Lucia et al., 2008; Derkacheva et al., 2013) and the EMF-complex, which suppresses precocious flowering by repressing *FT* and *AGL19* but which contributes also to repression of *FLC* (Yoshida et al., 2001; Schönrock et al., 2006a; Jiang et al., 2008). Because of the propeller-like structure of the WD40-domain, MSI1 and other MSI1-like proteins can possibly participate in additional chromatin-modifying complexes. Indeed, MSI1 was found to interact with LHP1 connecting plant PRC2 with LHP1 to establish repressive H3K27 methylation marks (Derkacheva et al., 2013). Further, MSI1 interacts with the CUL4-DDB1 complex and the Retinoblastoma-related protein to control imprinting in Arabidopsis (Jullien et al., 2008; Dumbliauskas et al., 2011). Chromatin-based mechanisms have recently emerged as a major means of control for many cellular processes including flowering time. In particular, the importance of chromatin-based regulation for control of *FLC* is well documented (Zografos and Sung, 2012).

Previously, we found that MSI1 represses drought stress responses (Alexandre et al., 2009) and is needed for timely flowering and for normal expression of *SOC1* (Bouveret et al., 2006). Here we demonstrate that MSI1 functions during floral transition by establishing normal expression of the flowering activator *CO* and subsequently of the florigen *FT* and *TSF*. We also show that delayed up-regulation of gene expression of the floral integrator genes correlates with the delay in flowering in a *msi1-*mutant. Our data suggest that MSI1 is needed for the efficient activation of *CO*, thus allowing full activity of the photoperiodic pathway for floral induction.

## Materials and methods

### Plant material

*Arabidopsis thaliana* ecotype Columbia (Col) was used throughout this study. T-DNA insertion lines *phyB* (SALK_022035, Mayfield et al., 2007; Ruckle et al., 2007), *pft1-2* (SALK_129555, Kidd et al., 2009), *flc-6* (SALK_41126, Schönrock et al., 2006a) and *msi1-5* (WiscDs Lox302B08) were obtained from NASC and confirmed by PCR. Seeds of *FRI*-Col, *esd1-10*, *ft-10* and *soc1-2* have been described (Lee and Amasino, 1995; Lee et al., 2000; Yoo et al., 2005; Martin-Trillo et al., 2006) and were kindly provided by J. Jarillo (FRI-Col, *esd1-10*), D. Weigel (*ft-10*), I. Lee (*soc1-2*), B. Ayres (*co-1*). The mutant *co-1* (accession La-0, Redei, 1962) was backcrossed into Col. The line *msi1-tap1* (accession Col) has been described before (Bouveret et al., 2006). Double mutants were identified among progeny of appropriate crosses by PCR with gene-specific primers (Supplementary Table 1).

To construct plants that ectopically overexpress *FT* (*35S::FT*), the full-length coding sequence was inserted into the binary destination vector pK7WG2 (Karimi et al., 2002) downstream of the cauliflower mosaic virus (CaMV) 35S promoter and transformed into *msi1-tap1* plants. Transformants were selected on kanamycin plates and genotyped by PCR. Hemizygous T2-generation plants of three independent T1 lines were analyzed for flowering time.

### Growth conditions and flowering time

Seeds were sterilized and plants were grown on Murashige and Skoog (MS) basal salt medium (Duchefa, Brussels, Belgium) after stratification at 4°C for 2–3 days. Plants were analyzed on plates or transferred to soil (“Einheitserde,” H. Gilgen optima-Werke, Arlesheim, Switzerland) 10 days after germination. Plants were kept in Conviron growth chambers with mixed cold fluorescent and incandescent light (130 μmol m^−2^ s^−1^, 21 ± 2°C) under (LD, 16 h light) or short-day (SD, 8 h light) photoperiods or were raised in green houses [LD: 14 h light, 19°C/10 h dark, 14°C; SD: 8 h light, 20°C/16 h dark, 20°C; supplemented with mercury vapor lamps (Sylvania Lighting S.A., Meyrin, Switzerland) to a maximum of 150 μmol m^−2^ s^−1^]. Flowering time was scored as described (Möller-Steinbach et al., 2010).

### RNA isolation and quantitative RT-PCR (qRT-PCR)

Total RNA was extracted as previously described (Hennig et al., 2003; Leroy et al., 2007; Alexandre et al., 2009). 1 μ g RNA treated with DNase I (Promega, Dübendorf, Switzerland) was transcribed into cDNA using a RevertAid First Strand cDNA Synthesis Kit (Fermentas, Nunningen, Switzerland) according to manufacturer's instructions. qRT-PCR with gene-specific primers (Supplementary Table 2) was performed on three technical replicates with the Fast Start Universal Probe Master (Rox) reagent and the Universal Probe Library set (UPL) (Roche Diagnostics, Rotkreuz, Switzerland) according to the manufacturer's instructions and results were normalized to *PP2A* as described (Exner et al., 2009). Shown is one of at least two independent biological experiments with similar results.

## Results

### MSI1 functions independently of light quality

Previously, we had reported that *MSI1* antisense lines and *msi1* mutants partially complemented with untagged *pMSI1::MSI1* or tagged *pMSI1::MSI1:TAP* constructs were late flowering (Bouveret et al., 2006). This suggests that undisturbed MSI1 levels are needed for normal flowering promotion. Homozygous *msi1* null mutants are lethal (Köhler et al., 2003; Guitton et al., 2004). Here, we analyzed heterozygous plants of the original *msi1-1* and a novel *msi1-5* allele and found that both flowered later than wild type under LD but not SD (Supplementary Figure 1). Similarly, *msi1-1*^−/−^ plants partially complemented with a *pMSI1::MSI1:HA* construct were late flowering (Supplementary Figure 1). Therefore the dose of *MSI1* is important for flowering time. The observation of a late flowering phenotype for *msi1*-mutants and transgenic lines motivated us to investigate the genetic pathway(s) in which MSI1 acts to affect flowering. Because the flowering delay was considerably more severe for the *msi1-1*^−/−^ *pMSI1::MSI1:TAP* (*msi1-tap*) line than for heterozygous *msi1* mutants, we used the *msi1-tap1* line in subsequent experiments. Unlike heterozygous *msi1* mutants, *msi1-tap1* flowered much later than wild type in SD (Bouveret et al., 2006). This is consistent with the generally milder late flowering phenotype of heterozygous *msi1* mutants. The normal flowering in SD may suggest that a single wild-type *MSI1* allele can largely suffice for normal *MSI1* functionin SD. It remains to be tested whether MSI1 requirements are lower in SD or whether a potential compensatory mechanism can more efficiently up-regulate the remaining *MSI1* allele in SD than in LD.

We then tested a potential function of MSI1 in the light quality pathway, which functions through phytochromes and independently of the circadian system. PHYB, PHYD, and PHYE repress *FT* expression and therefore flowering, with PHYB having the major role in this process (Kim et al., 2008). The PHYB target PFT1 was proposed to directly activate both, *CO* and *FT* expression, while it simultaneously acts as negative regulator of phytochrome signaling by inactivation of PHYB protein (Cerdan and Chory, 2003; Wollenberg et al., 2008). Null alleles of *phyB* and *pft*, which are early and late flowering, respectively, were introduced into the *msi1-tap1* background and flowering time was analyzed in LD. The loss of PHYB in the *phyB msi1-tap1* double mutant led to flowering with 15 rosette leaves (RL) in LD, which was intermediate between the *phyB* single mutant (4 RL) and *msi1-tap1* (19 RL, Table 1). This result suggests an additive interaction between *MSI1* and *PHYB*. The loss of PFT1 in the *pft1 msi1-tap1* double mutant resulted in a synergistic delay in flowering (41 RL) compared to the *pft* and *msi1-tap1* single mutants (14 and 17 RL respectively, Table 1), suggesting likewise independent effects of *MSI1* and *PFT1* in flowering promotion but likely on the same common targets. Together, these results propose a function of MSI1 independent of the light quality pathway in floral induction.

**Table 1:** **Flowering time of double mutants of *msi1-tap1* and different flowering time mutants in LD**.

		**Rosette leaves**	**Days to bolting**
1	Col	12.1 ± 0.4	28.8 ± 0.6
	*msi1-tap1*	19.2 ± 0.7	36.5 ± 0.6
	*phyB*	4.3 ± 0.2	18.9 ± 0.3
	*phyB msi1-tap1*	15.4 ± 0.6	30.9 ± 0.7
2	Col	9.1 ± 0.2	26.5 ± 0.2
	*msi1-tap1*	17.4 ± 0.6	33.7 ± 0.5
	*pft1*	14.4 ± 0.3	32.7 ± 0.5
	*pft1 msi1-tap1*	41.3 ± 3.7	59.8 ± 3.4
3	Col	7.3 ± 0.4	20.8 ± 0.9
	*msi1-tap1*	19.6 ± 1.4	40.1 ± 1.3
	Col *FRI*	72.7 ± 3.1	79.4 ± 2.7
	Col*FRI msi1-tap1*	84.6 ± 3.1^a^	143.8 ± 12.2^a^
	*flc*	6.2 ± 0.1	21.0 ± 1.1
	*flc msi1-tap1*	16.9 ± 0.8	34.3 ± 0.5
4	Col	10.4 ± 0.5	27.8 ± 0.5
	*msi1-tap1*	19.9 ± 0.5	36.9 ± 0.5
	*esd1*	5.0 ± 0.1	24.0 ± 0.0
	*esd1 msi1-tap1*	11.9 ± 0.4	33.3 ± 0.3
5	Col	7.1 ± 0.4	23.9 ± 0.6
	*msi1-tap1*	16.5 ± 0.8	39.0 ± 0.6
	*fca*	68.6 ± 2.5	75.9 ± 4.0
	*fca msi1-tap1*	57.0 ± 1.1^a^	289.6 ± 41^a^

Shown are mean value ± SE (n ≥ 14).

a Analysis of flowering time was stopped when ~1/3 of the plants had died before bolting. Number of rosette leaves and days until death or termination of the experiment are shown.

### MSI1 functions independently of the floral repressor *FLC* on flowering

**Figure 1:**
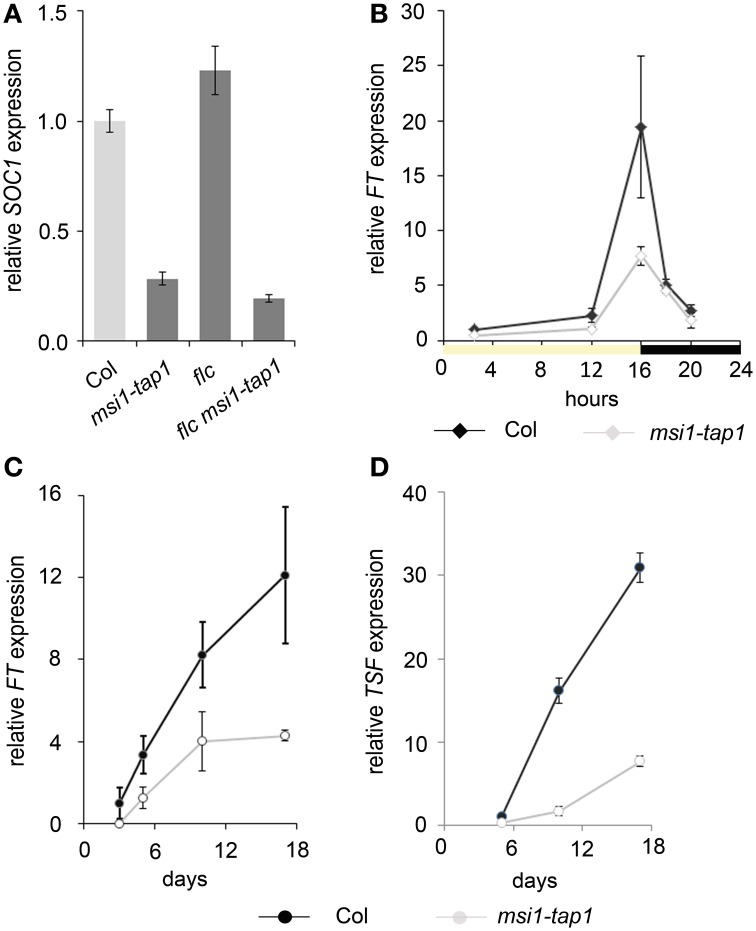
**Expression of *SOC1, FT,* and *TSF* is reduced in *msi1-tap1* in LD. (A)** *SOC1*-expression in 9-day-old seedlings of Col (light gray), *msi1-tap1*, *flc,* and *flc msi1-tap1* (dark gray) in LD at EOD (end of day). **(B)** Diurnal expression pattern of *FT* in 10-day-old seedlings. X-axis represents hours after start of the light period (yellow box—light period, black box—dark period). **(C,D)** Gene expression kinetics of *FT* **(C)** and *TSF* **(D)** in Col and *msi1-tap1* at the end of the day (EOD) at 5, 10, and 17 days after germination (DAG). Values are relative expression ± SE (*n* = 3). Black lines, Col; gray lines, *msi1-tap1*.

Next, we investigated the potential role of the flowering repressor *FLC* in MSI1 effects on flowering time. *FLC* is one of the main regulators of flowering time in Arabidopsis, and altered flowering time is often caused by altered *FLC* expression. Consistent with earlier observations (Bouveret et al., 2006), analysis of *flc msi1-tap1* double mutants suggested that late flowering of *msi1-tap1* was independent of *FLC,* as evident from the largely unaffected late flowering of *msi1-tap1 flc* plants (Table 1). Previously, we observed reduced *SOC1* expression in *msi1-tap1* (Bouveret et al., 2006). Here, we tested whether the reduced expression of *SOC1* was independent of *FLC*. *SOC1* expression in the double mutant was as low as in *msi1-tap1*, suggesting that the *flc* mutation could not lift *SOC1* repression in *msi1-tap1* plants (Figure 1A). Because MSI1 was recently shown to be involved in *FLC* control as part of plant PRC2 complexes (De Lucia et al., 2008; Derkacheva et al., 2013), we performed additional genetic tests of a potential role of *FLC* in *msi1-tap1* late flowering. The active *FRI*-allele of the late flowering Arabidopsis accession San Feliu (Sf2) crossed into Columbia (Col *FRI*), was introgressed into *msi1-tap1*. As previously reported (Lee et al., 1993; Clarke and Dean, 1994), Col *FRI* flowered very late, possibly due to high *FLC* expression (Table 1). Col *FRI msi1-tap1* plants flowered much later (85 RL) than either parent (73 and 20 RL for Col *FRI* and *msi1-tap1*, respectively). Some of the Col *FRI msi1-tap1* plants were not able to flower at all and died without completing their life cycle. This additive delay in flowering suggests an independent role of *MSI1* and *FRI* in flowering. Previously, we found a strongly synergistic interaction between *MSI1* and *FVE* (Bouveret et al., 2006). *FVE* is part of the autonomous pathway, which represses *FLC*, and genes in this pathway were grouped in two epistasis groups. While *FVE* represents one of the two groups, *FCA* is a gene from the second group. Here, we tested the genetic interaction between *MSI1* and *FCA*. The *fca msi1-tap1* double mutants were extremely delayed in flowering. They ceased to produce leaves without starting to bolt or flower leading to a smaller rosette leave number than for *fca*. After an extended period of developmental inactivity they eventually died (Table 1). The strongly synergistic interaction suggests that *MSI1* and *FCA* do not function in the same genetic pathway to control flowering time.

Another activator of *FLC* is *EARLY IN SHORT DAYS1* (*ESD1*, also known as *SUPPRESSOR OF FRIGIDA 3* and *ACTIN RELATED PROTEIN 6*). Mutations in *ESD1* hasten flowering through reduced *FLC* expression in LD and SD (Martin-Trillo et al., 2006; Choi et al., 2007; Lazaro et al., 2008). An early flowering *esd1* mutant allele was crossed into *msi1-tap1*. In LD, the *esd1 msi1-tap1* double mutant flowered intermediate (12 RL) to both parents *esd1* and *msi1-tap1* (5 and 20 RL, respectively) disclosing an additive effect between ESD1 and MSI1 on flowering (Table 1). These data suggest that *ESD1* and *MSI1* function in separate genetic pathways. Together, these results firmly established that *MSI1* can function independently of *FLC* to affect flowering time in LD.

### Changes in *MSI1* lead to reduced levels of *FT* and *TSF*

FT and its homolog TSF are activators of *SOC1* (Yamaguchi et al., 2005; Yoo et al., 2005). Increased *FT* expression was found in *msi1-tap1* suppressor mutants, which rescued the *msi1-tap1* late flowering phenotype (Exner et al., 2009, 2010). In LD-grown wild-type Arabidopsis, CO activates *FT* at the end of the day (Suarez-Lopez et al., 2001; Yanovsky and Kay, 2002). To test whether the diurnal rhythm of *FT* was affected in *msi1-tap1*, *FT* expression was profiled throughout the light-dark cycle in seedlings (Figure 1B). In wild type, *FT* had its expression peak toward the end of the light and beginning of the dark period as previously reported. The *FT* expression in *msi1-tap1* followed the same pattern as in wild type, but expression values were lower, especially at the end of the day (EOD), when expression of *FT* was reduced by up to 50%. Additionally, we tested whether MSI1 affected the temporal activation of *FT* or its homolog *TSF* (Figures 1C,D). Under LD conditions, *FT* and *TSF* levels increased steadily in wild type between 3 and 17 days. In *msi1-tap1*, *FT* transcripts started to accumulate similarly to wild type but the increase was much slower leading to considerably reduced *FT* levels. The accumulation of *TSF* transcripts was even stronger reduced in *msi1-tap1* leading to 70% lower levels than in wild type at 17 days after germination. These results demonstrate that normal MSI1-function is needed for typical activation of *FT* and its homolog *TSF* in LD.

**Figure 2:**
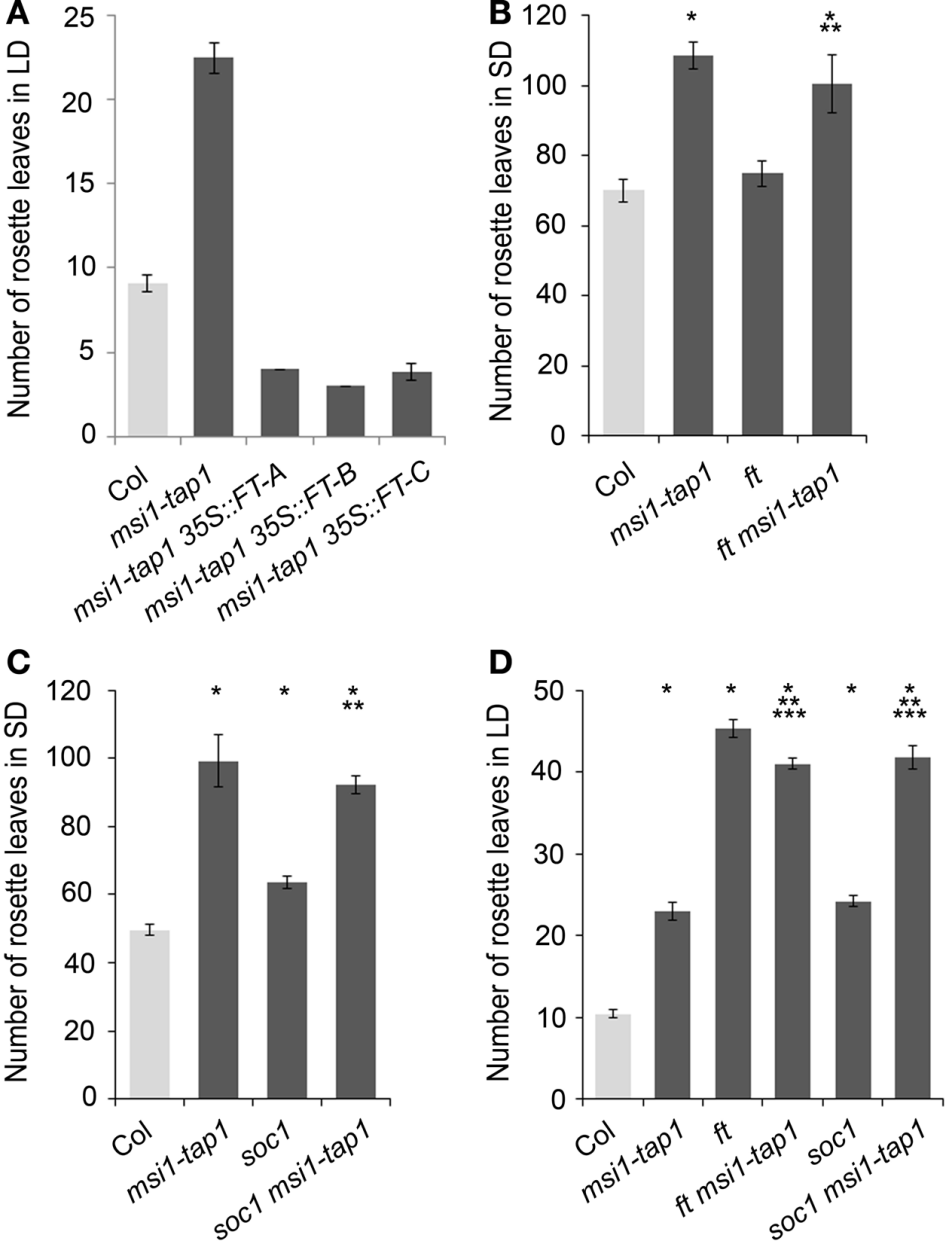
**Flowering time analysis of *msi1-tap1* and double mutants. (A)** Flowering time of Col, *msi1-tap1* and *msi1-tap1 35S::FT* lines in LD. The *35S::FT* suppresses the late flowering of *msi1-tap1*. **(B)** Flowering time of Col, *msi1-tap1, ft,* and *ft msi1-tap1* in SD. **(C)** Flowering time of Col, *msi1-tap1, soc1*, and *soc1 msi1-tap1* in SD. **(D)** Flowering time of Col, *msi1-tap1, ft, ft msi1-tap1, soc1* and *soc1 msi1-tap1* in LD. Light gray bars represent wild type Col. Shown are means ± SE of rosette leaves (*n* ≥ 14). Significance of difference was tested using *t*-tests. Asterisks denote differences that were significant at *p* < 0.05 to WT (^*^), single mutant (^**^), or *msi1-tap1* (^***^).

To test whether higher *FT* expression can be sufficient to suppress the late flowering phenotype of *msi1-tap1*, a *35S::FT* transgene was introduced into *msi1-tap1*. The *FT* over-expression caused extremely early flowering (Figure 2A), which is consistent with the notion that reduced *FT* expression contributed to the late flowering of *msi1-tap1*.

To substantiate that delayed activation of *FT* and therefore of *SOC1* was responsible for the late flowering of *msi1-tap1*, a *ft* mutant allele was crossed into *msi1-tap1* for flower time measurements. The double mutant *soc1 msi1-tap1*, which was already described in LD before (Bouveret et al., 2006), was included into the analysis (Figures 2B–D; Supplementary Figure 2). Under SD conditions, *ft* flowered similar to wild type, and the *ft msi1-tap1* line flowered similar to *msi1-tap1*, confirming that *FT* does not play a major role under these conditions (Figure 2B) (Yanovsky and Kay, 2002; Corbesier et al., 2007). In contrast to *FT, SOC1* functions in induction of flowering in SD (Borner et al., 2000) and the *soc1* single mutant flowered later than wild type (Figure 2C). While the *soc1 msi1-tap1* line needed longer until flowering than either parent, it produced a similar number of RL as the *msi1-tap1* parent suggesting that delayed activation of *SOC1* contributes at least partially to the late flowering of *msi1-tap1* in SD. Thus, during flowering induction in SD, MSI1 and SOC1 appear to function partially in the same genetic pathway.

Under LD conditions, both *ft* and *soc1* flowered later than wild type consistent with their roles in photoperiodic flowering (Borner et al., 2000). The double mutant *soc1 msi1-tap1* exhibited an additive late flowering phenotype (42 RL) compared to the *msi1-tap1* and *soc1* parents (23 and 24 RL, respectively, Figure 2D) confirming earlier results (Bouveret et al., 2006). The *ft msi1-tap1* line flowered with 42 RL similar to the *ft* parent (45 RL) supporting the notion that reduced *FT* expression is the main reason for late flowering of *msi1-tap1* in LD (Figure 2D). In summary, *MSI1* affects full activation of *FT*, *TSF* and *SOC1* expression to promote timely flowering.

### MSI1 function is connected to the photoperiod pathway

**Figure 3:**
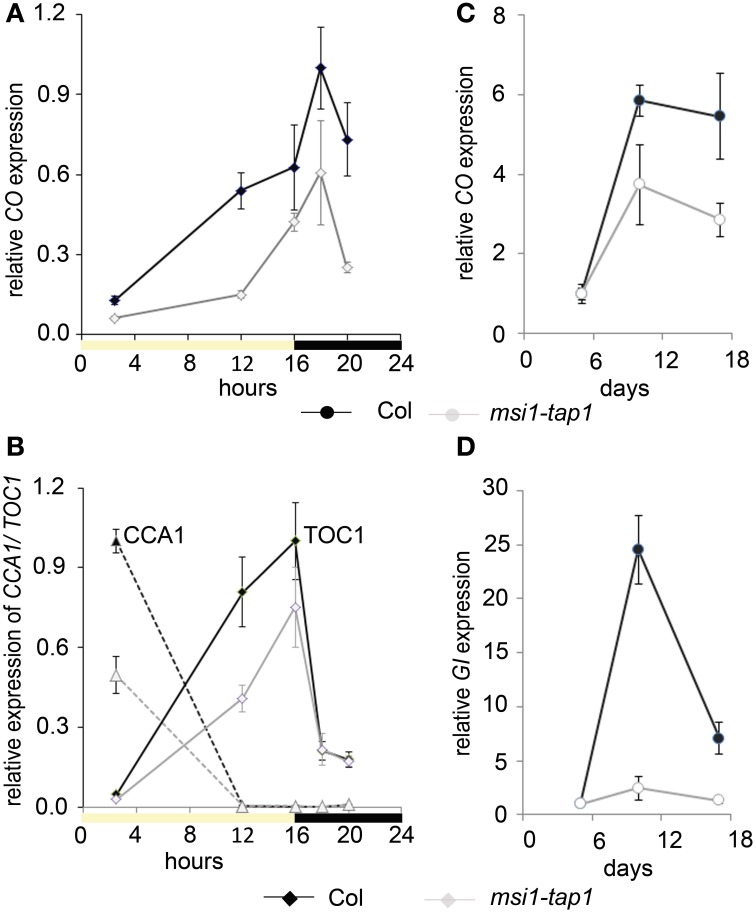
**Gene expression of *CO* and *GI* is changed in *msi1-tap1* in LD.** Diurnal gene expression of *CO* **(A)** and *CCA1* and *TOC1* **(B)** in 10-day-old seedlings in LD. X-axis represents hours after start of the light period (yellow box—light period, black box—dark period). Black lines, Col; gray lines, *msi1-tap1*. **(C,D)** Gene expression kinetics of *CO* **(C)** and *GI* **(D)** in *msi1-tap1* at EOD at 5, 10, and 17 DAGs. Values are relative expression ± SE (*n* = 3).

Because CO is a main activator of *FT, SOC1*, and *TSF* (Suarez-Lopez et al., 2001; Hepworth et al., 2002; Yamaguchi et al., 2005), we asked whether reduced expression of *FT, SOC1,* and *TSF* in *msi1-tap1* was caused by defects in *CO* regulation. *CO* is under strong circadian and diurnal control (for review see Searle and Coupland, 2004), and *CO* expression in *msi1-tap1* was tested throughout an entire light-dark cycle. This experiment revealed that *CO* expression was considerably lower in *msi1-tap1* than in wild type (Figure 3A). The *CO* expression in wild type showed the previously reported peak toward the end of the day and beginning of the dark. Similarly, this expression pattern was observed for *msi1-tap1* suggesting that diurnal regulation was not grossly altered. This conclusion was supported by normal diurnal cycling of *CCA1* and *TOC1*, two components of the central circadian oscillator. However, under the tested conditions, *CCA1* and *TOC1* showed lower amplitudes of peak expression values in *msi1-tap* (Figure 3B). Further, we analyzed the *CO* transcript levels at different developmental time points until 17 days after germination (Figure 3C). Under our conditions, *CO* increased steadily in wild type during 10 days after germination. In *msi1-tap1*, *CO* transcripts started to accumulate similarly to wild type but the increase was slower leading to considerably reduced *CO* levels. Together, the expression data suggest the hypothesis that MSI1 affects expression of *FT*, *TSF*, and *SOC1* and flowering time in LD via *CO*.

**Figure 4:**
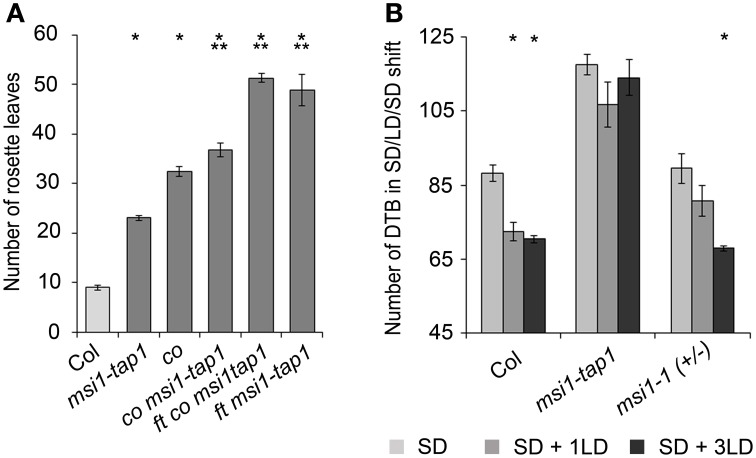
**MSI1 is needed for normal sensitivity of the photoperiod. (A)** Flowering time of Col, *msi1-tap1, co, co msi1-tap1*, *co ft msi1-tap1,* and *ft msi1-tap1* in LD expressed in mean number of RL ± SE (*n* ≥ 14). Significance of difference was tested using *t*-tests. Asterisks denote differences that were significant at *p* < 0.05 to WT (^*^) or *co* (^**^). **(B)** Flowering time of Col, *msi1-tap1*, and heterozygous *msi1-*1in SD and after single LD treatments. Plants were grown continuously in SD (light gray bars) or exposed to 1 or 3 LD (16 h light/8 h dark; dark gray and black columns, respectively) at 45 DAG. LD treatments were performed by extension of the light period and shortening of the following dark period. Flowering time is expressed in DTB ± SE (*n* ≥ 14). Asterisks denote significant differences to the corresponding SD-only control (*t*-test, *p* < 0.05).

To test genetically whether reduced *CO* expression was responsible for delayed flowering of *msi1-tap1*, a *co* mutant allele was introduced into the *msi1-tap1* line. Consistent with earlier findings (Koornneef et al., 1991; Robson et al., 2001), the *co* mutant was late flowering in LD. While *msi1-tap1* delayed flowering substantially in the *CO* wild-type background, it only slightly delayed flowering of a *co* mutant (Figure 4A) suggesting that late flowering in *msi1-tap1* is caused mainly by effects on *CO*. The similar flowering time of *ft msi1-tap1* and the *ft co msi1-tap1* triple mutant (Figure 4A) further supported the notion of an epistatic genetic interaction between *MSI1* and *CO*.

Because GIGANTEA (GI) is a major activator of *CO* expression (Imaizumi et al., 2005; Sawa et al., 2007), we tested whether *GI* expression was altered in *msi1-tap1*. At 5 d after germination, when *CO* levels did not differ between WT and *msi1-tap1*, *GI* was also not affected (Figure 3D). In contrast, at 10 d and 17 d, not only *CO* but also *GI* expression was substantially reduced in *msi1-tap1*.

Together, these data suggest that *MSI1* acts on flowering in response to the photoperiod through *GI* and *CO* on *FT*.

### MSI1 is needed for normal sensitivity of the photoperiod pathway

To further test the importance of MSI1 in the photoperiodic pathway, we performed a SD-LD-SD shift experiment. In SD, *FT* is only very weakly activated due to immediate destabilization of CO protein after synthesis, and flowering is very much delayed. A brief LD experience can be sufficient to activate *FT* and induce flowering if the photoperiodic pathway functions normally (Corbesier et al., 1996; King et al., 2008). We cultivated plants under SD conditions interrupted by 1 or 3 days of LD at 45 days after germination and measured flowering time (Figure 4B). Under these conditions, Col was highly sensitive to the additional LD exposures, and flowering was accelerated by about 3 weeks. In contrast, the effect on flowering time of *msi1-tap1* was minor and not statistically significant. Heterozygous *msi1-1* mutants reacted like wild type to three additional LDs but showed a reduced response to a single additional LD (Figure 4B). Together, these results demonstrate that MSI1 is needed for normal sensitivity of the photoperiodic pathway.

## Discussion

In plants, flowering at the right time is determined by several endogenous and external signals. One of the genes affecting flowering is *MSI1*. Late flowering was observed in lines expressing either tagged (TAP-, GFP-, HA) or untagged MSI1 under a 2 kb *MSI1*-promoter fragment in a *msi1* mutant background (Bouveret et al., 2006; Alexandre et al., 2009; this work). In addition, *MSI1*-antisense lines and heterozygous *msi1* mutants were late flowering, together establishing that normal *MSI1* function is needed for normal timing of flowering. The *MSI1-TAP* construct did not affect flowering in WT plants nor did these plants in any other way differ from WT. Similarly, when a *35S::MSI1* construct was introduced into *msi1-tap1*, the late flowering was suppressed (Bouveret et al., 2006). Therefore, we consider it unlikely that the late flowering of *msi1-tap1* plants was caused by a dominant negative effect of the fusion protein. Here, we used this line as a tool to dissect the function of the essential *MSI1* gene in flowering time control.

One signal affecting flowering is light quality, which gives information about competition by neighboring plants and is sensed mainly by phytochromes, in particular PHYB (for review see Thomas, 2006). In light-quality sensing, PHYB functions via PFT1 both to activate *CO* and to activate *FT* in a *CO*-independent way (Cerdan and Chory, 2003; Iñigo et al., 2012a,b). In addition, PHYB has also functions in photoperiod sensing (for review see Thomas, 2006). Genetic interaction analysis between *PHYB* and *MSI1* showed an additive flowering time phenotype suggesting a function of *MSI1* independent from the light quality pathway to promote flowering. Similarly, *PFT1* and *MSI1* did not show an epistatic interaction, suggesting that both genes function in distinct genetic pathway. The finding that *PFT1* and *MSI1* showed a tendency for a synergistic genetic interaction with a greater than additive flowering delay, is consistent with the notion that both genes commonly affect *CO* and *FT* in flowering time control. Interestingly, *MSI1* and *PFT1* both affect not only flowering time but also drought stress responses (Alexandre et al., 2009; Elfving et al., 2011). Because PFT1 is a subunit of the Mediator complex (Bäckström et al., 2007), future studies should aim to test whether MSI1 and Mediator share direct targets.

In Arabidopsis, FLC is a major repressor of flowering and mutants deficient in *FLC* repression are often late flowering. *FLC* is repressed both by vernalization and also by the autonomous pathway to allow flowering even without vernalization (Baurle et al., 2007). Previously, it was shown that MSI1 functions both in the major *FLC*-dependent vernalization pathway and in a *FLC*-independent vernalization pathway that regulates *AGL19* (Schönrock et al., 2006a; De Lucia et al., 2008; Derkacheva et al., 2013). Here we find that MSI1 can affect flowering independent of vernalization and of *FLC*. This conclusion is based on genetic interaction studies between (i) *MSI1* and *FLC*, (ii) *MSI1* and *FRI*, an *FLC*-activator (Michaels and Amasino, 2001), (iii) *MSI1* and *FCA* or *FVE*, two *FLC* repressors from the autonomous pathway, and (iv) *MSI1* and *ESD1*/*SUF3*/*ARP6*, a *FLC*-activator and putative subunit of the SWR1 complex (Martin-Trillo et al., 2006; Choi et al., 2007; Lazaro et al., 2008).

In summary, MSI1 affects flowering time independent of the light quality pathway and of *FLC*.

The late flowering of *msi1-tap1* could be explained as a consequence of reduced expression of *FT*, *SOC1,* and *TSF*. Genetic interaction analysis showed epistatic effects of *FT* with *MSI1*, demonstrating that MSI1 functions through the main flowering time integrators to promote flowering. *FT*, which is a major activator of *SOC1*, is in turn activated by CO in the photoperiod pathway to promote flowering in LD (Putterill et al., 1995). Strict diurnal regulation of CO protein levels is controlled by several complex pathways coupled to the core circadian oscillator and light conditions (for review see Andres and Coupland, 2012). *CO* is repressed in the morning by PHYB and activated in the evening by *GI*. The diurnal expression pattern of *CO* appeared not significantly altered in *msi1-tap1*, where *CO* still shows an expression peak late in the day. The level of detectable *CO* mRNA, however, was substantially reduced in *msi1-tap1*. The lower abundance of *CO* mRNA is associated with reduced *GI* expression. Together, reduced *GI* expression in *msi1-tap1* could cause the reduced *CO* expression that in turn delays activation of *FT* and eventually *SOC1*and could explain the delay in flowering.

Although GI is thought to function mainly by directly activating *CO*, GI can also directly activate *FT* and accelerate flowering in the absence of CO (Sawa and Kay, 2011). Notably, *msi1-tap1* did not cause any further delay of *ft* mutants but could slightly delay *co* mutants. These observations are consistent with a model in which reduced *GI* expression in *msi1-tap1* does not only affect flowering via reduced *CO* levels but also directly via compromising *FT* activation.

Here, we studied the role of MSI1 in flowering under LD conditions and identified its function upstream of the photoperiodic *CO*-*FT* module. However, MSI1 has also a function for flowering under SD conditions, and this is independent of *FT*. Flowering in SD depends on *SOC1* (Borner et al., 2000). We found not only that *SOC1* expression is reduced in *msi1-tap1* plants but also that *MSI1* and *SOC1* show a genetic interaction in SD suggesting that under these conditions *MSI1* affects flowering by contributing to normal *SOC1* expression. It remains to be tested which other flowering time genes are affected by MSI1 and contribute to the late flowering phenotype of *msi1-tap1* in SD.

**Figure 5:**
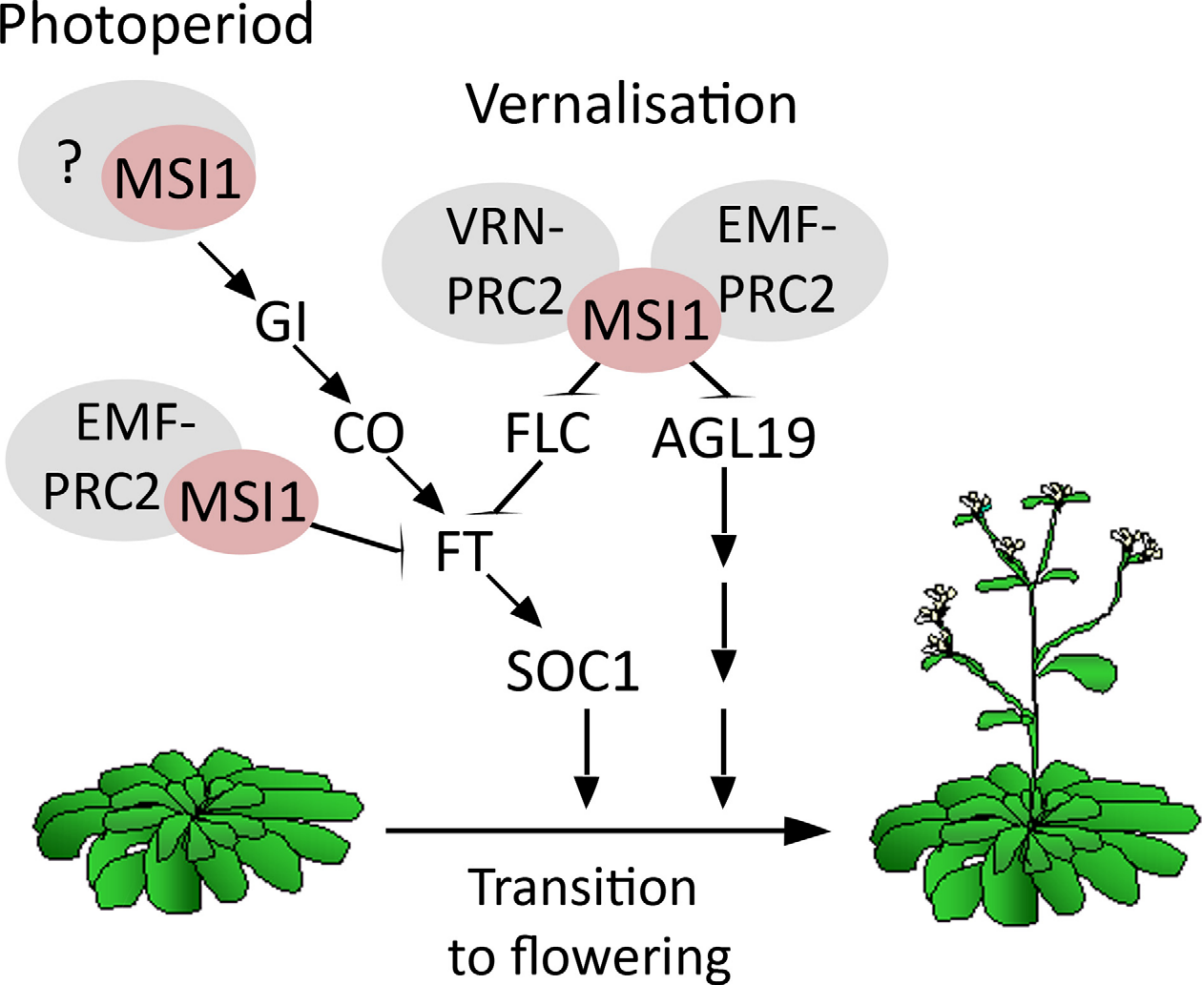
**Model of MSI1 function in flowering time control.** Scheme of the different pathways MSI1 is acting in to affect flowering time. First, MSI1 represses flowering via its functions in the EMF-PRC2-complex to represses *AGL19* prior to vernalization and *FT* prior to photoperiodic activation (Schönrock et al., 2006a; Jiang et al., 2008). Second, MSI1 favors flowering via its function in the VRN-PRC2 complex to repress *FLC* after vernalization (De Lucia et al., 2008; Derkacheva et al., 2013). Third, MSI1 affects flowering through the photoperiodic *GI*-*CO*-*FT* pathway.

This work and earlier studies have established that MSI1 affects flowering in multiple pathways (Figure 5). First, MSI1 represses flowering via its functions in the EMF-PRC2-complex to represses *AGL19* prior to vernalization and *FT* prior to photoperiodic activation (Schönrock et al., 2006a; Jiang et al., 2008). Second, MSI1 favors flowering via its function in the VRN-PRC2 complex to repress *FLC* after vernalization (De Lucia et al., 2008; Derkacheva et al., 2013). Here, we have shown that MSI1 affects flowering in a third way—by contributing to *CO* expression MSI1 allows to rapidly respond to photoperiod. The relative importance of these diverse functions will depend on conditions, such as LD vs. SD or with or without vernalization treatment. Given that the histone adaptor MSI1 may be part of additional complexes, it is possible that MSI1 affects flowering in even other ways.

## Author contributions

Yvonne Steinbach conceived and carried out the experiments and analyzed the data. Yvonne Steinbach and Lars Hennig planned the study and wrote the manuscript. All authors read and approved the final manuscript.

## Conflict of interest statement

The authors declare that the research was conducted in the absence of any commercial or financial relationships that could be construed as a potential conflict of interest.

## Supplementary material

Supplementary Figure 1 **Characterization of a new *msi1* allele and the transgenic *msi1-HA* lines. (A)** Insertion site of the WiscDsLox302B08 T-DNA in the second exon of *MSI1*-gene locus (*AT5G58230*). **(B)** Scheme of the *pMSI1::MSI1:HA* transgene containing the MSI1 cDNA fused to a triple HA-tag at the C-terminus under control of 2 kb of the *MSI1*-promoter. **(C)** Flowering time of heterozygous *msi1-*1 and *msi1*-5 mutants in LD (gray bars) and SD (dark bars) in RL ± SE (*n* ≥ 14). **(D)** Flowering time of *msi1-HA* lines expressed in RL ± SE (*n* ≥ 14). **(E)** Seed abortion rate in heterozygous *msi1-*5 mutants and homozygous *msi1-HA* lines. Siliques of *msi1-*5 contained about 50% normal and 50% aborted seeds, similar to the embryo lethal phenotype of the *msi1-1* mutant (Köhler et al., 2003). The *pMSI1::MSI1-HA* construct can rescue the seed abortion phenotype similar to *pMSI1::msi1-tap1* (Bouveret et al., 2006).

Supplementary Figure 2 **Flowering time of msi1-tap1 and double mutants. (A-C)** Flowering time in LD of **(A)** Col, *msi1-tap1, soc1, soc1 msi1-tap1*. **(B)** Col, *msi1-tap1, ft, ft msi1-tap1*. **(C)** Col, *msi1-tap1, co, co msi1-tap1, ft co msi1-tap1 and ft msi1-tap1*. **(D,E)** Flowering time in SD of **(D)** Col, *msi1-tap1, soc1, soc1 msi1-tap1,* **(E)** Col, *msi1-tap1, ft, ft msi1-tap1*. Values are shown in mean DTB ± SE (*n* ≥ 14).. Significance of difference was tested using *t*-tests. Asterisks denote differences that were significant at *p* < 0.05 to WT (^*^), of the double mutants to the appropriate single mutant (^**^) or msi1-tap1 (^***^).

Supplementary Table 1 **Primers used for genotyping and cloning**.

Supplementary Table 2 **qRT-PCR primers used in this study**. Shown are the forward and reverse primers with the appropriate Universal probe library (UPL) (Roche) probe number.
