# Supplementary material for: Arabidopsis MSI1 functions in photoperiodic flowering time control
Source: Front Plant Sci. 2014 Mar 7;5:77. doi: 10.3389/fpls.2014.00077 (PMC3945484; doi:10.3389/fpls.2014.00077)
Supplement: Supplementary Table 1 — Primers used for genotyping and cloning. [file DataSheet1.ZIP › 78897_Steinbach_Supplementary Table 1.pdf]

Supplementary Table 1 Primers used for genotyping and cloning.

Allele	Forward primer	Reverse primer	T-DNA primer
CO/ co-1	CACCGGATCCATATGTTGAAACA AGAGAGTAACG	GTTGACTCCGGCACAACAC	Digest with *Bfa*I[1]
ESD1/ esd1-10	AATCGTCTACGACACCGAGC	ATTCCAGCCTGCAGATTTAGG	P745
FT	GGTGGAGAAGACCTCAGGAA	GGTTGCTAGGACTTGGAACATC	
ft-10		TATAACAGGAAACCTAGTCCTGCTC	GK8409
MSI1	GATTCTAGGGTTATAACGAGG	GATGCCATGCAACATCTTCCAC	
msi1-1		GATGCCATGCAACATCTTCCAC	LB3
msi1-5		CCGTTCAGAAGATGATTCTCG	P745
PHYB/ phyB	GTTCCCAAGTCGACTAAACCG	AAGGGTCCTGATGCTTTAACC	LBb1
PFT1/ pft1-2	TCTGAATAGGTCCATTGGCTG	TTGATGGTGGGAAACTTGATC	LBb1
Col *FRI*	ATTTGCTGGATTTGATAAGG	TTATACCATCAAGCTTATCG	
LBb1	GCGTGGACCGCTTGCTGCAACT		
LB3	TAGCATCTGAATTTCATAACCAATCTCGATACAC		
GK8409	ATATTGACCATCATACTCATTGC		
P745	AACGTCCGCAATGTGTTATTAAGTTGTC		
For cloning			
FT cDNA	CACCATGTCTATAAATATAAGAG ACCC	CTAAAGTCTTCTTCCTCCGCA	
MSI1 cDNA	GTTCAAGCAGGGTCATTTGC	GTCTCGTTCTTTGGGTTCCA	
3xHA	GTACCATGGGGCAGGTTACCCAT ACGTTCC	CTAACTAGTAGTGGACGCCTCTAG AGGAACG	

[1]The *co* mutant lost one restriction site for *Bfa*I in the *CO*-gene. To distinguish between the *co*mutant and wild-type version, PCR-fragments were digested with *Bfa*I.
