# Supplementary material for: Arabidopsis MSI1 functions in photoperiodic flowering time control
Source: Front Plant Sci. 2014 Mar 7;5:77. doi: 10.3389/fpls.2014.00077 (PMC3945484; doi:10.3389/fpls.2014.00077)
Supplement: Supplementary Table 1 — Primers used for genotyping and cloning. [file DataSheet1.ZIP › 78897_Steinbach_Supplementary Table 2.pdf]

Supplementary Table 2: qRT-PCR primers used in this study. Shown are the forward and reverse primers with the appropriate Universal probe library (UPL) (Roche) probe number.

Gene	Forward primer	Reverse primer	UPL[1] probe no.
CCA1	AAAAAGTGTCGCATCCTGAGA	GAACAGTTGTCTTCCTGCAGAGT	#124
CO	GCCTACTTGTGCATGAGCTG	GTTTATGGCGGGAAGCAAC	#53
FT	GGTGGAGAAGACCTCAGGAA	GGTTGCTAGGACTTGGAACATC	#138
GI	TTTCCGATGGTGTAGTGGTG	TTGAAGGCATCAGTTGAGGA	#67
SOC1	GAGTTTTGCCCCTCACCA	CAAACCCTTTTAGCCAATCG	#143
TOC1	AGTCACCAGGAAAATGAGTGG	AAGACCACCATCACGAGCAT	#68
TSF	TGGAGGAGACGACTTCAGAAA	GCTTGGACTCGGCACATC	#138
PP2A[2]	GGAGAGTGACTTGGTTGAGCA	CATTCACCAGCTGAAAGTCG	#82

1) UPL probe - Universal Probe Library probe
2)Reference gene
